# Rapid patient-specific FEM meshes from 3D smart-phone based scans

**DOI:** 10.1088/1361-6579/ad26d2

**Published:** 2024-02-28

**Authors:** Ethan K Murphy, Joel Smith, Michael A Kokko, Seward B Rutkove, Ryan J Halter

**Affiliations:** 1 Thayer School of Engineering, Dartmouth College, Hanover, NH 03755, United States of America; 2 Department of Neurology, Beth Israel Deaconess Medical Center (BIDMC), Boston, MA 02215, United States of America; 3 Harvard Medical School, Boston, MA 02115, United States of America; 4 Geisel School of Medicine, Dartmouth College, Hanover, NH 03755, United States of America

**Keywords:** electrical impedance tomography, 3D scanning, finite element meshes, subject specific meshing

## Abstract

*Objective.* The objective of this study was to describe and evaluate a smart-phone based method to rapidly generate subject-specific finite element method (FEM) meshes. More accurate FEM meshes should lead to more accurate thoracic electrical impedance tomography (EIT) images. *Approach.* The method was evaluated on an iPhone^®^ that utilized an app called Heges, to obtain 3D scans (colored, surface triangulations), a custom belt, and custom open-source software developed to produce the subject-specific meshes. The approach was quantitatively validated via mannequin and volunteer tests using an infrared tracker as the gold standard, and qualitatively assessed in a series of tidal-breathing EIT images recorded from 9 subjects. *Main results.* The subject-specific meshes can be generated in as little as 6.3 min, which requires on average 3.4 min of user interaction. The mannequin tests yielded high levels of precision and accuracy at 3.2 ± 0.4 mm and 4.0 ± 0.3 mm root mean square error (RMSE), respectively. Errors on volunteers were only slightly larger (5.2 ± 2.1 mm RMSE precision and 7.7 ± 2.9 mm RMSE accuracy), illustrating the practical RMSE of the method. *Significance.* Easy-to-generate, subject-specific meshes could be utilized in the thoracic EIT community, potentially reducing geometric-based artifacts and improving the clinical utility of EIT.

## 1. Introduction

Electrical impedance tomography (EIT) is a novel technology exhibiting promise in numerous clinical applications including cancer, stroke, and hemorrhage detection (Poplack *et* *al* 2007, Murphy *et al* 2017, Avery *et al* 2018, Murphy *et al* 2022). However, its predominant and most clinically developed application is in monitoring regional lung ventilation and for regional pulmonary function testing in patients with chronic lung diseases (Frerichs *et al* 2017, Leonhardt and Lachmann 2012). EIT is safe, low-cost, portable, and capable of providing real-time, long-term monitoring of patients, making it an attractive technique for study.

In these real-time applications, difference imaging is used, which produce images showing the change over time in conductivity relative to a baseline measurement. Difference imaging can significantly reduce the impact of systematic noise. Slight boundary shape errors can be tolerated (Grychtol *et al* 2012), but generally, improper domain modelling can yield significant artifacts (Murphy and Mueller 2009, Boyle *et al* 2017). These artifacts can make images hard to interpret and overall reduce the clinical utility of EIT. Prior efforts to improve boundary information or reduce these artifacts have included (1) recording CTs of the thorax and segmenting the boundary (Zhao *et al* 2014), (2) incorporating the boundary shape into the EIT inverse problem (Soleimani *et al* 2006, Nissinen *et al* 2011, Brazey *et al* 2022), (3) using EIT belts with bend sensors or accelerometers (de Gelidi *et al* 2018, Darma *et al* 2020), and most-recently (4) video-based scanning of the thorax (Dussel *et al* 2022). CT imaging of the electrodes is best, assuming metal artifacts can be properly removed, but is impractical or too costly in many situations. Solving for the boundary shape via the impedance data appears very promising based on the published works, but the approaches do not appear to be validated in human subject studies as of yet. The accelerometer-based belt also appears quite promising but does require manufacturing of custom belts. This study utilizes the Face ID sensor from a standard iPhone to collect a 3D scan of the thorax with belt and custom, open-source software (Murphy 2023a, 2023b) to rapidly produce patient-specific meshes. Given the ubiquity of iPhones, this approach can provide EIT researchers a fast and accessible way to obtain subject-specific meshes. Comparisons to Dussel *et al* (2022) will be discussed.

## 2. Methods

This section describes the iPhone-based subject-specific mesh construction process, which includes the belt setup, scanning process, construction of surface mesh and electrode labeling, and 3D finite element method (FEM) mesh construction. The software is available on Github, which can be accessed via Zenodo (Murphy 2023b, 2023c), and the corresponding data of this study is available (Murphy 2023a). Validation tests are subsequently described, including (1) comparisons between an iPhone and commercial infrared tracking system on both a mannequin and human subjects and (2) real-world evaluation of tidal-breathing EIT images generated from subject-specific meshes on 9 subjects enrolled in a study evaluating EIT as a surrogate measure for pulmonary function tests (PFT).

### 2.1. iPhone-based subject-specific mesh construction

**Figure 1.**
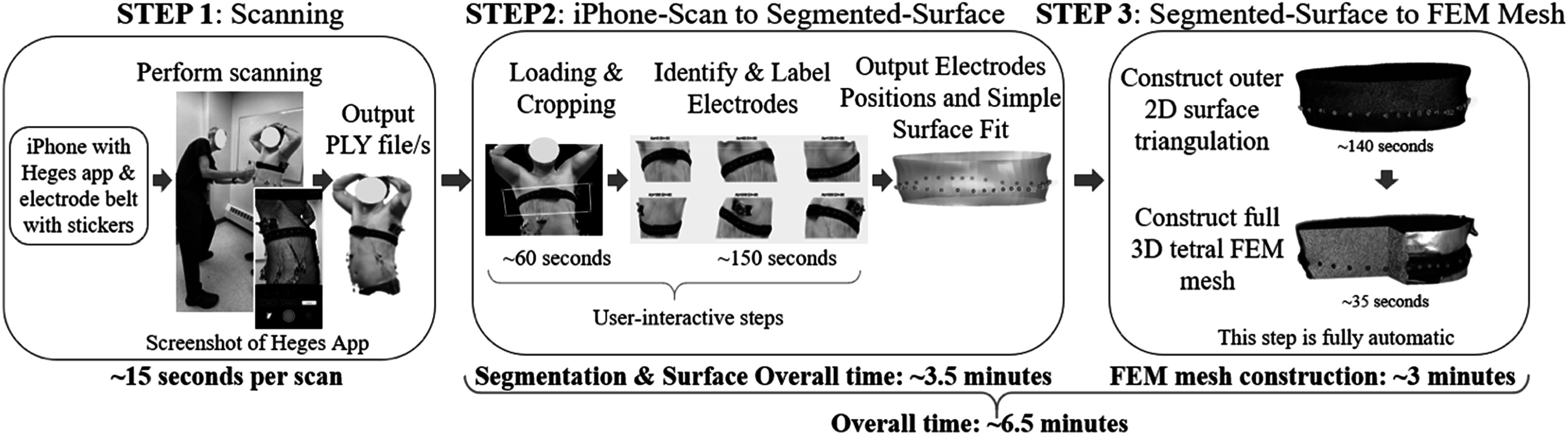
Workflow of the iPhone scanning and processing to output subject-specific meshes.

The main components of the approach include the custom-belt design, iPhone scanning, and image processing and mesh construction software. An overview of the workflow (figure 1) shows the three main steps (1: Scanning, 2: iPhone-scan to Segmented-Surface, and 3: Segmented-surface to FEM mesh), and additionally, notes approximate times and where user interaction is needed.

#### 2.1.1. Belt design

**Figure 2.**
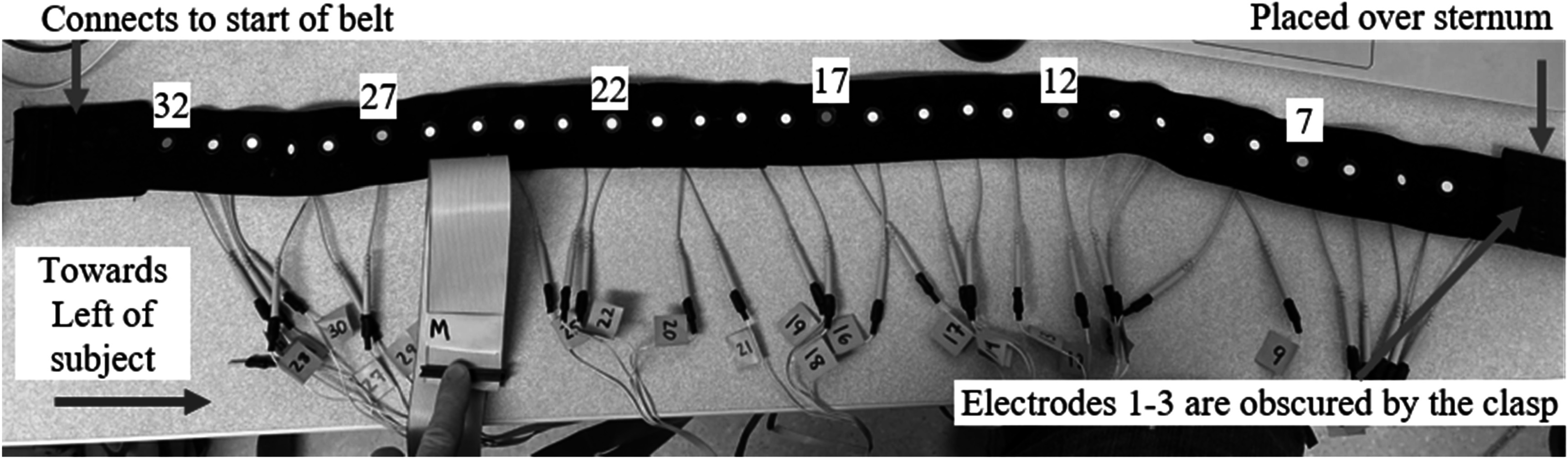
One of three belts used in the study. It has 32 reusable electrodes connected to an elastic, cloth belt with three sets of clasps sewn into belt allowing for small variations in thorax size. Stickers are placed on the opposite side of all the electrodes with six colored stickers placed on particular electrodes (7 = cyan, orange = 12, 17 = red, 22 = yellow, 27 = green, and 32 = pink). The known colored locations enable fast labeling of the electrodes.

As part of an ongoing study exploring EIT as a surrogate measure for PFTs, specifically focused on amyotrophic lateral sclerosis (ALS) patients, we designed three different sized custom 32-eletrode belts (figure 2 shows a photo of the medium belt). The electrodes are reusable over-the-counter (OTC) Patient’s Choice^®^ Silver 0.8′′ Round Tan Tricot electrodes. This electrode type has recently been discontinued, and updated belts use 1 cm Red Dot ECG electrodes. Fiducial stickers were placed on the outside of the belt opposing each electrode. Six of the fiducials were marked with specific colors to aid in the fast labeling of the electrodes.

#### 2.1.2. iPhone scanning

The approach utilizes the Heges App with the Face ID sensor on an iPhone to collect 3D scans. An iPhone 12 Pro was used in the study, but other iPhones including versions 11 or newer, and models X, XR, and XS, could also theoretically be used. There are multiple 3D scanning iPhone apps available. Heges was preferred as it yielded good performance while also ensuring all scanned data stayed local to the phone, minimizing privacy concerns. Scans were recorded using 1 mm resolution and maximum range. The output of each scan is a PLY file that consists of a surface triangulation with RGB color information at each node.

#### 2.1.3. STEP 1: scanning

**Figure 3.**
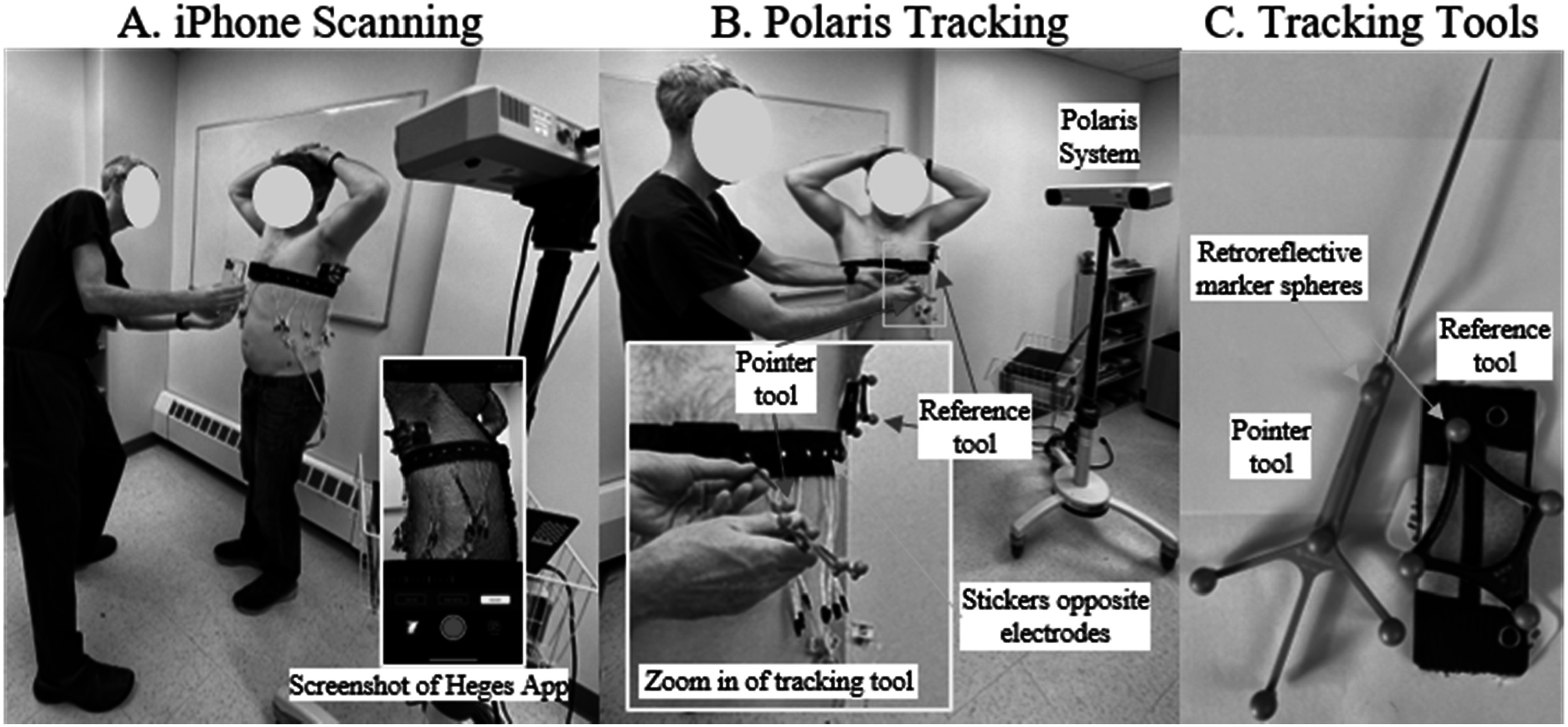
Photos of the A. iPhone scanning on a volunteer (showing a screenshot from the Heges app) and B. Polaris recording electrode positions. The Polaris tracking involves the base system (labeled Polaris system) and a pointer and reference tool (see C for large views of the tools and retroreflective marker spheres).

The scanning consists of (1) the subject standing in an open area, and (2) a researcher slowly walking around the subject with the phone 1–2 feet (∼30–60 cm) from the subject scanning the belt. It is important to have sufficiently bright and uniform lighting. The scanning started focused on the sternum, proceeded around the subject, and finished pointing at the sternum. The subjects were asked to remain still, while breathing normally, with their arms over their heads, so as to maintain a clear view of the belt. In the volunteer (validation) scans, subjects were asked to do a breath hold or minimize breathing dynamics. Scans were collected in approximately 15 s, and at least two scans were taken per subject. The app provides feedback via vibration, informing the user to slowdown, and by providing real-time updates of the scan on the iPhone screen (figure 3(A)). After the scan is recorded, the researcher can view the scan to ensure it appears qualitatively adequate. If scans appeared of poor quality or a full-scan failed, then additional scans were collected. Poor quality scans are generally due to an artifact in the scan, such as the body or arm of the person performing the scan, or moving the iPhone too quickly around the subject, and qualitatively inferior scans do not connect to form a contiguous thorax.

#### 2.1.4. STEP 2: iPhone-scan to segmented-surface

The first set of software (Murphy 2023a) implemented in MATLAB imports the PLY file and, with some user input, produces a smooth surface triangulation with electrodes labeled and numbered. The steps are as follows: (1) loading and cropping the surface, (2) clicking on electrodes (fiducial markers: 6 colored stickers followed by the remaining white stickers), (3) leveling the scan, and (4) producing a simple, smooth fit to the surface—which we refer to as the *surface fit*. Steps 1 and 2 require input from the user. Step 1 asks the user to define 4 points in a view of the scan to crop and only retains the region of interest (electrodes/belt and sufficient space above and below the belt). Step 2 involves clicking on electrodes following the instructions provided in the title of the MATLAB figures. Leveling is performed by finding a best-fit plane to the segmented electrodes, e.g. (Hartov *et al* 2010). The surface fit produced combines a Fourier series (*x*/*y*-directions) with radial-basis-functions (RBF) (*z*-direction), i.e. it extends the common Fourier series for 2D-chest approximations into 3D (Murphy *et al* 2019).

#### 2.1.5. STEP 3: segmented-surface to FEM mesh

The second set of software (Murphy 2023b), also implemented in MATLAB, takes the segmented surface as input and automatically (1) produces a new surface mesh with encoded electrodes via distmesh (Persson and Strang 2004) and (2) constructs a 3D FEM mesh using gmsh (Geuzaine and Remacle 2009). Essentially the same steps as described in Murphy *et al* (2019) are used. However, the code has been improved in terms of speed, robustness, and has been incorporated into Murphy (2023b). The open-source software distmesh and gmsh are required for the software package to work. The only required modifications to the custom software package is setting paths to the user’s local copy of distmesh and gmsh. Empirically determined, hardcoded h-values for elements near the electrodes (0.5 mm) and in the background of the domain (5 mm) are provided. These values could be adjusted to produce a more or less dense mesh.

### 2.2. Validation tests

**Figure 4.**
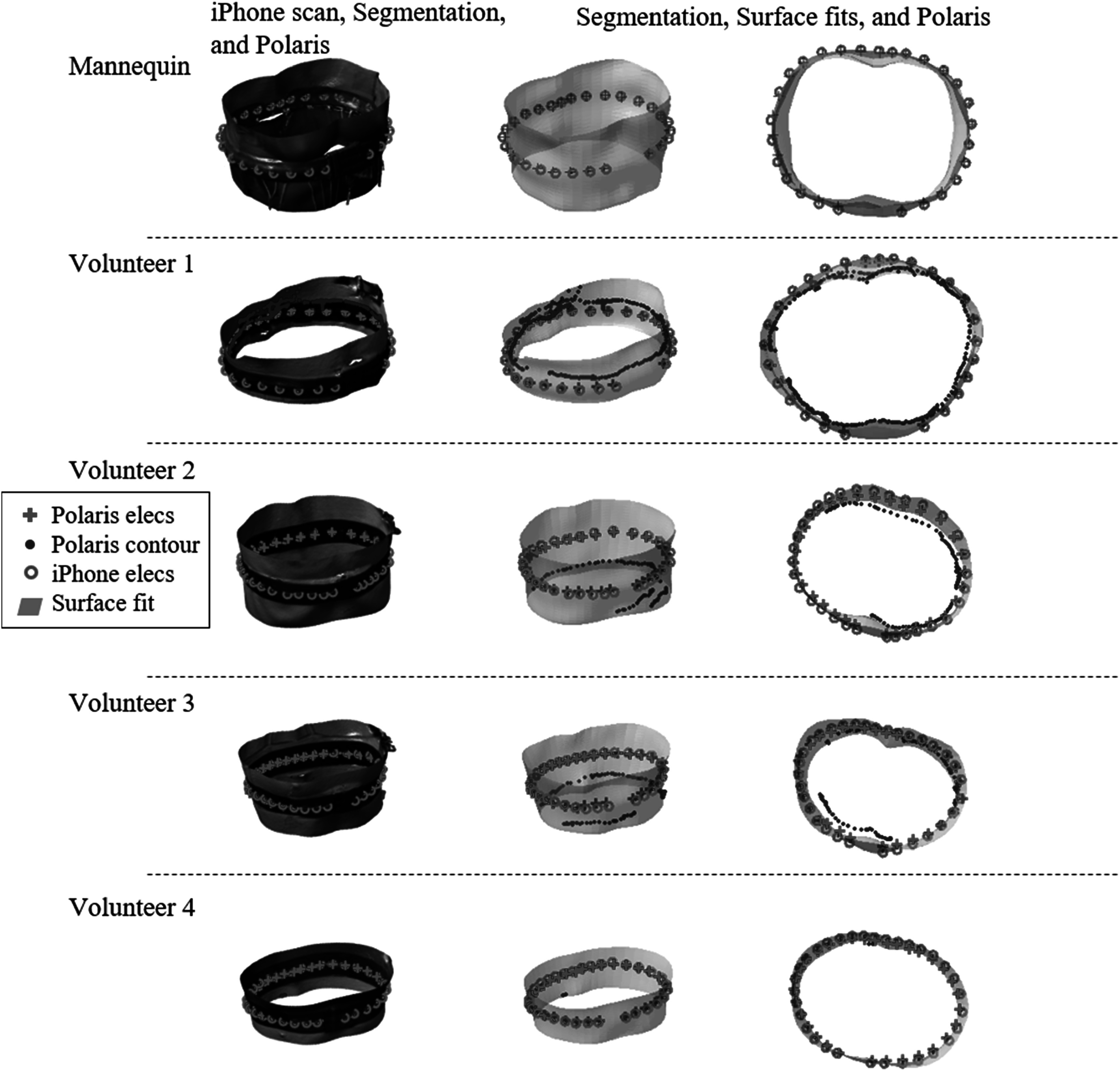
Overall mannequin and volunteer data collected. Column 1 shows the iPhone scans (colored surfaces) along with the Polaris and iPhone-based electrode locations and the Polaris contours. The contours show locations traversed by the pointer tip (figure 3(C)) across the subjects’ thoraxes. Columns 2 and 3 provide two views of the surface fit to the iPhone scan and the same electrode and contour data.

Validation tests evaluating the precision (i.e. reproducibility) and accuracy of the 3D scans were performed on (1) a mannequin thorax and (2) four volunteers. In these tests, 3D scans were captured using the iPhone, and additionally an NDI Polaris Hybrid P4 infrared tracker (Northern Digital, Inc., Waterloo, ON) was used to collect electrode positions and thorax contours (see figure 3 for an overview of the measurement setup). The Polaris system tracks the position and orientation of a pivot-calibrated Bucholz Freehand 960–556 pointer tool (Medtronic, PLC, Minneapolis, MN, see figure 3(C)), which has five retroreflective marker spheres. Probe tip positions were reported with respect to a four-marker Bucholz Freehand 9732236 reference tool (see figure 3(C)) that was attached to the mannequin and volunteers to minimize errors due to subject motion. Root mean square (RMS) differences and errors of electrodes were computed from the iPhone-to-iPhone, Polaris-to-Polaris, and iPhone-to-Polaris scans. For the mannequin tests, three iPhone scans and three sets of Polaris electrode positions were collected; whereas for the volunteers, four iPhone scans, two sets of Polaris electrode positions, and one Polaris contour were collected per volunteer. Electrode errors were additionally evaluated in terms of position on the scan and qualitatively in terms of histograms. Accuracy of the surfaces (cropped iPhone scan and surface fit) were evaluated by comparing those outputs to contours traced on the thorax of the volunteers with the tracked pointer, using the Polaris system outputs as ‘ground truth’. The contours were collected on the front and back of the volunteers above the belt and represent a relatively small sampling of the entire thorax (see blue curves in figure 4).

### 2.3. Electrical impedance tomography data and imaging

EIT data was collected from 9 consented subjects participating in an institutional review board (IRB) approved study (Dartmouth Hitchcock Medical Center, 02000545) investigating the use of EIT as a surrogate measure for PFTs on ALS patients as compared to healthy subjects. EIT data was collected using the 32-electrode belt and the SenTec EIT Pioneer System (SenTec AG, Landquart, Switzerland) with an injection current of 3 mA, signal frequency of 195 kHz, 4-skip injection pattern, and ∼48 Hz imaging. EIT images were produced of tidal breathing (differencing the average maximum inhale minus the maximum exhale) from recordings of 30 s of tidal breathing from each subject. The same impedance noise filtering described in Munir *et al* (2020) was used here. EIT images were produced by a one-step Gauss–Newton approach with standard Tikhonov regularization and normalized voltage data. Images were produced with a generic chest mesh and an iPhone-based subject specific one. Images were evaluated qualitatively. The Tikhonov parameters were tuned over all subjects for the generic and the subject-specific meshes.

## 3. Results

### 3.1. Validation tests

The iPhone scan and surface generation were validated using recordings on a mannequin and four volunteers utilizing a Polaris infrared tracker. Probe tip calibration RMS error was 0.25 mm, and practically it is expected to have an accuracy of approximately 1 mm within its imaging domain given a 305 mm probe tip offset (Wiles *et al* 2004). The axis ratios of the mannequin and four volunteers were 1.1, 1.1, 1.2, 1.5, 1.4, respectively, and their perimeters were 104.8, 111.4, 92.9, 80.0, 77.5 cm, respectively. The volunteers were composed of 2 males (volunteers 1–2) and 2 females (volunteers 3–4) with an average age, height, and weight of 35.3 ± 9.2 year (average ± standard deviation (SD)), 174.6 ± 5.6 cm, and 70.5 ± 12.0 kg, respectively. As the validation test focused on surface measurements below the nipple line, there should be minimal effect of gender (not recorded) or sex of the subjects, and consequently, errors with respect to sex were not analyzed.

#### 3.1.1. Mannequin tests

**Figure 5.**
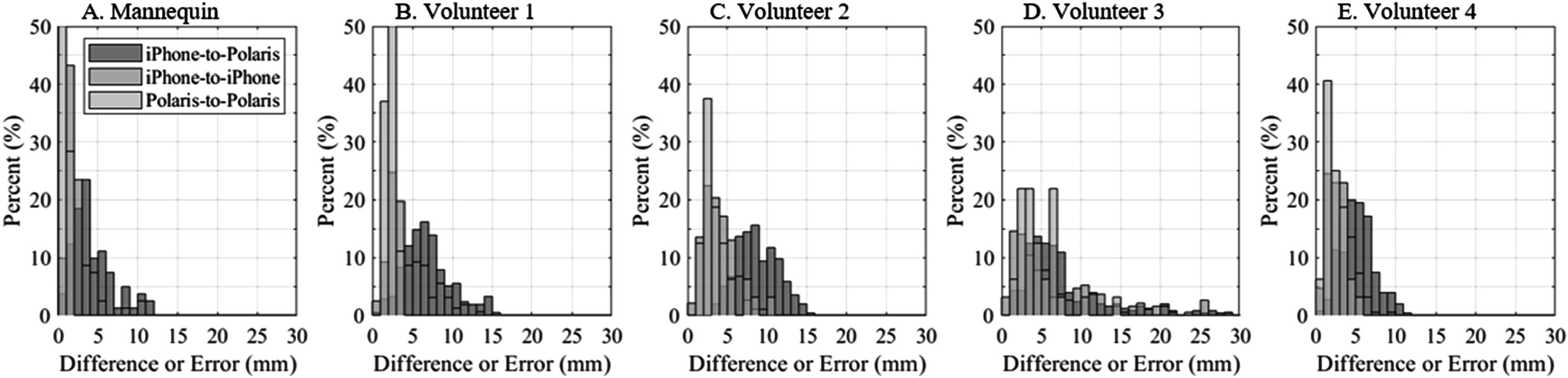
Histogram of differences and error from the iPhone and Polaris electrode position comparisons for the A. Mannequin and B–E. Volunteers, respectively. The histograms provide an additional view of the distribution beyond what is expressed by the average, SD and maxima.

Precision and accuracy of the iPhone and Polaris electrode positions for the mannequin tests are reported table 1. Precision was assessed by comparing combinations of the three successive within-subject scans (3 comparisons from scans 1 and 2, scans 1 and 3, and scans 2 and 3). The RMS differences were 3.2 ± 0.4 mm and 0.9 ± 0.1 mm for the iPhone and Polaris, respectively, and maximum differences were 9.9 ± 1.1 mm and 1.7 ± 0.2 mm for the iPhone and Polaris, respectively. Each RMS value was calculated across 27 electrode position pairs (5 were occluded due to the buckle), and the averages and SDs reported in the final column of table 1 are based on the 3 previous columns (*n* = 3). It is presumed that the Polaris error is larger than the expected 1 mm (see above) accuracy due to variability in the exact location the tool was pointed due to user error. That is, we needed to precisely point to the center of the 12 mm sticker and keep it still during the recording. The iPhone had average and maximum precision errors 2.3 and 8.2 mm worse than the Polaris tracker. Interestingly, the iPhone precision is very similar to its accuracy, 3.2 versus 4.0 mm RMS and 9.9 versus 10.0 mm maximum errors. Qualitatively, the iPhone and Polaris data (top row of figure 4) shows very good agreement between techniques. Histograms of differences or error of the iPhone and Polaris data (figure 5(A)) show that the iPhone-to-iPhone and iPhone-to-Polaris errors are more centered around the average values and the maxima appear qualitatively to be outliers.

**Table 1.** Mannequin validation data.

Precision		Scans 1 and 2	Scans 1 and 3	Scans 2 and 3	Average
iPhone-to-iPhone	Elec RMS (mm)	3.4	2.7	3.4	3.2 ± 0.4
	Elec Max (mm)	10.7	10.2	8.7	9.9 ± 1.1
Polaris-to-Polaris	Elec RMS (mm)	1.1	0.8	0.9	0.9 ± 0.1
	Elec Max (mm)	1.9	1.6	1.6	1.7 ± 0.2
Accuracy		Scans 1	Scans 2	Scans 3	Average
iPhone-to-Polaris	Elec RMS (mm)	4.3	3.9	3.8	4.0 ± 0.3
	Elec Max (mm)	11.8	6.5	11.7	10.0 ± 3.1

#### 3.1.2. Volunteer tests

The second set of validation tests was performed using a similar protocol on four volunteers, where four iPhone scans and two sets of Polaris data were collected on each volunteer. Precision and accuracy from these tests are given in table 2 for the electrode positions, and additionally errors associated with the iPhone surface, surface fit, and traced contours of the surface. Average RMS precisions were 5.2 ± 2.1 mm and 3.6 ± 1.5 mm for the iPhone and Polaris, respectively, and maximum differences were 10.2 ± 4.5 mm and 8.5 ± 4.8 mm. In comparison to the mannequin tests, the iPhone RMS errors are 2 mm larger and the maximum differences are essentially identical, whereas the Polaris RMS and maximum differences errors are both larger (2.7 mm and 6.8 mm, respectively). The increased errors are not surprising given breathing and possible motion of subjects. The iPhone accuracy RMS errors and maximum differences were 7.7 ± 2.9 mm and 12.4 ± 5.6, which are 2.5 mm and 2.2 mm worse than the precision error, respectively. RMS values were calculated across 27 electrode position pairs for volunteer 1 (5 were occluded due to the buckle) and over all 32 electrodes for volunteer 2–4. The number of samples used in average and SD calculations in table 2 are as follows: the average column is based on the 4 volunteer average results (*n* = 4), contour/surfaces results involve 100–1000 s of points (*n* > 100), precision calculations for each iPhone-to-iPhone utilized 6 scan comparisons (*n* = 6), and accuracy calculations utilized 8 iPhone-scan-to-Polaris comparisons (*n* = 8). Qualitatively the scans appear similarly accurate to the mannequin scans (see figure 4). Error histograms (figures 5(A)–(E)) show the larger errors in the volunteer versus mannequin tests and the larger errors for volunteer 3.

**Table 2.** Volunteer validation data.

Precision		Vol 1	Vol 2	Vol 3	Vol 4	Average
iPhone-to-iPhone	Elec RMS (mm)	5.2 ± 1.3	4.1 ± 0.8	8.2 ± 5.6	3.4 ± 0.6	5.2 ± 2.1
	Elec Max (mm)	11.1 ± 2.9	7.0 ± 1.3	16.1 ± 11.7	6.5 ± 1.4	10.2 ± 4.5
Polaris-to-Polaris	Elec RMS (mm)	2.2	4.3	5.4	2.6	3.6 ± 1.5
	Elec Max (mm)	3.8	10.9	14	5.3	8.5 ± 4.8
iPhone (PLY)-to-surface fit		1.1 ± 0.8	0.8 ± 0.7	0.7 ± 0.6	0.6 ± 0.3	0.8 ± 0.7
Accuracy		Vol 1	Vol 2	Vol 3	Vol 4	Average
iPhone-to-Polaris	Elec RMS (mm)	7.3 ± 1.9	9.0 ± 1.5	8.8 ± 4.6	5.6 ± 0.8	7.7 ± 2.9
	Elec Max (mm)	11.6 ± 3.6	12.8 ± 1.7	15.8 ± 10.0	9.5 ± 1.4	12.4 ± 5.6
	Contour-to-PLY	8.2 ± 4.4	10.8 ± 2.8	7.2 ± 6.0	4.1 ± 0.3	8.5 ± 4.7
	Contour-to-Surface fit	11.5 ± 4.5	12.6 ± 2.8	9.3 ± 5.2	5.5 ± 2.1	11.1 ± 4.5

#### 3.1.3. Surfaces

The surface errors give information on (1) precision through comparison of the iPhone (PLY) surface to the surface fit and (2) accuracy through comparison of the Polaris contours (see blue curves in figure 4) to the PLY and surface fit. The surface fit appears to give a good approximation of the PLY file, 0.8 ± 0.7 mm difference, which is notable as the surface fit is defined by only 175 parameters while the cropped PLY files average ∼350 K nodes. The accuracy surface errors appear to be reasonably consistent with the accuracy RMS errors of electrode positions, i.e. 7.7 mm for the electrodes compared to 8.5 and 11.1 mm for the PLY and surface fit, respectively.

### 3.2. Tidal breathing subject evaluation

In addition to the volunteer tests, the iPhone scans were collected and evaluated on tidal breathing recordings of subjects from a pulmonary function study to assess the real-world impact on difference EIT imaging. There were 13 scans analyzed on a total of 9 subjects where iPhone and EIT data were collected from a study investigating EIT as a surrogate measure for PFTs on ALS patients. The subjects age, height, and weight were 63.7 ± 11.1 years, 75.2 ± 16.8 kg, and 170.7 ± 6.6 cm, respectively. There were 6 males and 3 females (gender was not recorded), of which, 8 were healthy controls and 1 an ALS patient. The belts were placed just below the nipple line, as high as possible below the bra for female subjects. As the tidal images are assessed qualitatively, no explicit analysis was done across sexes. Although the anatomical cross-sections should be essentially the same, the belt may have been placed slightly lower on female subjects.

#### 3.2.1. Tidal breathing EIT images

**Figure 6.**
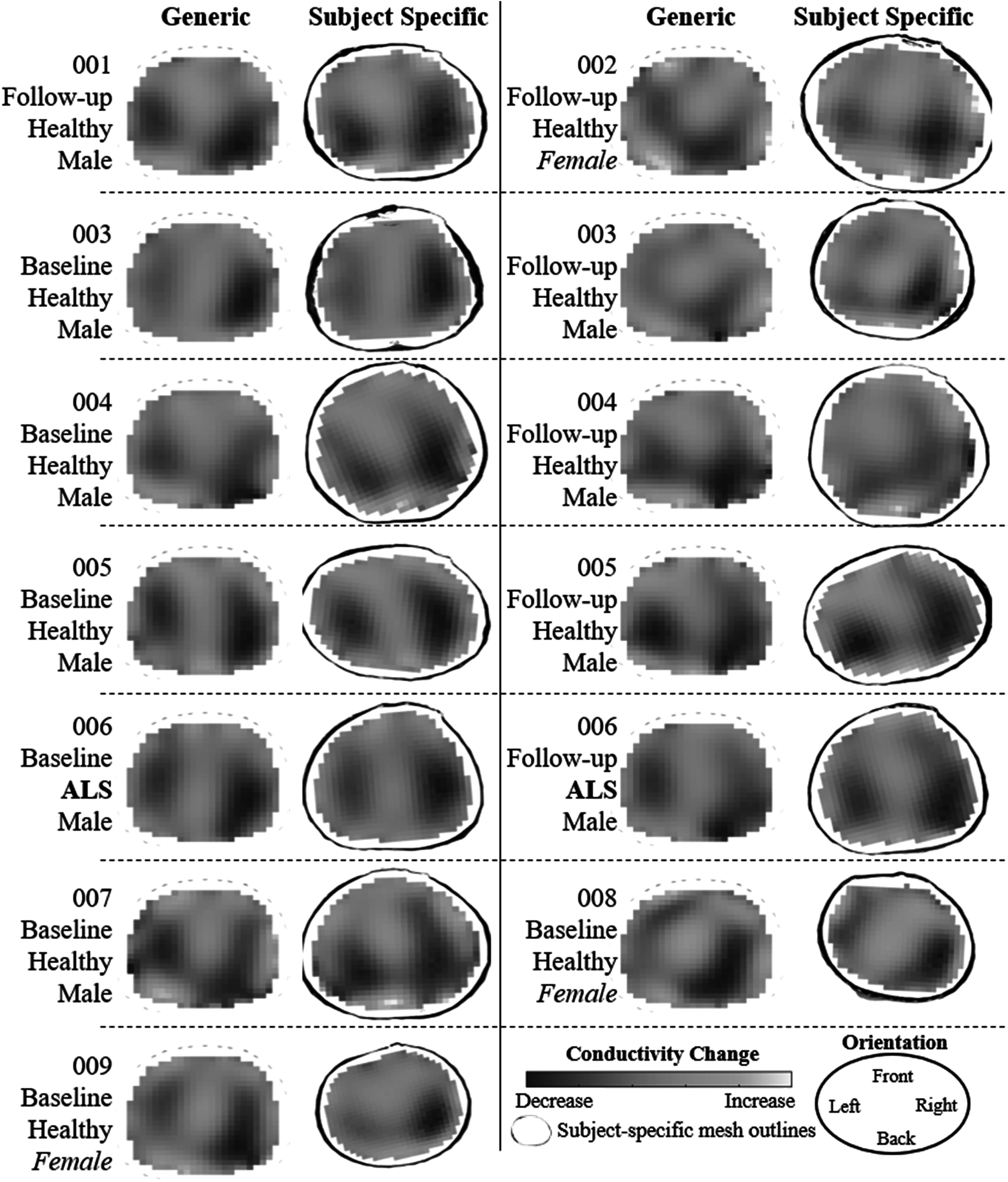
EIT difference reconstructions of average maximum inhale and exhale from tidal breathing for all available iPhone scans using either a generic mesh or subject specific mesh. The data collection type (baseline or follow), subject status (healthy or ALS) and sex are noted.

The 13 tidal images reconstructed using a generic and subject-specific mesh (figure 6) qualitatively show the expected two blue regions corresponding to a decrease in conductivity in the inflated lung regions. Comparing generic to subject-specific images, one can clearly see that the lungs (dark blue regions) are anatomically more accurately orientated in the subject-specific meshes. While all cases exhibit this improvement to some degree, the most dramatic examples of this are observed in 002 and 007. Excluding the rotation issues of the generic meshes, artifacts appear relatively comparable between approaches, e.g. 001, 002, and 007. The subtle differences illustrate how difference imaging is good at removing systematic noise. However, the small differences in positions of the lungs using the generic versus the subject-specific meshes could affect how clinicians would interpret these images. Further analysis of these images is deemed outside the scope of this study.

#### 3.2.2. Processing times

The processing time needed to generate a subject-specific FEM mesh from the iPhone acquired PLY file was on average 380.8 ± 95.2 s (6.3 ± 1.6 min). The minimum and maximum overall times were 3.9 and 8.7 min, respectively. We note that the Heges processing times are essentially negligible. The user-interactive loading/crop and electrode identification steps took on average 60.3 ± 42.5 and 146.6 ± 44.8 s, respectively. The remaining steps were fully automatic: surface fit, outer surface mesh (distmesh) and 3D FEM mesh (gmsh) took 0.6 ± 0.2, 139.0 ± 57.4, and 34.3 ± 8.1 s, respectively. The FEM meshes have on average 381 K nodes, 1.85 M elements, and their extent in the *z*-direction was 13.1 ± 3.5 cm.

## 4. Discussion

This study utilized an iPhone to rapidly produce subject-specific FEM meshes (∼6 min from PLY files to a full 3D FEM mesh) with high precision (3.2 ± 0.4 mm RMSE) and accuracy (4.0 ± 0.3 mm RMSE) on mannequin validation tests and only slightly larger errors on volunteer data (5.2 ± 2.1 mm RMSE precision and 7.7 ± 2.9 mm RMSE accuracy, *n* = 4). Given that there may have been motion due to breathing or position shifts, an increase of 1.2 mm and 3.7 mm appears to indicate the method is practical and can yield small electrode localization errors. Here we discuss (1) the clinical impact, (2) alternative scanners, belts, and scenarios, and (3) a comparison to other scanning approaches and boundary studies.

### 4.1. Clinical impact

A lingering question, that has not been resolved by the tidal breathing analysis performed here, is determining the clinical impact of having subject-specific meshes (either of the current quality or potentially improved quality through the above strategies). This is viewed as beyond the scope of this study. However, more accurate modeling will produce more accurate EIT images, which could increase the anatomical interpretability and confidence in the resulting images.

### 4.2. Alternative scanners

In principle, the posted software should be compatible with any modern handheld 3D scanner, be it a separate scanner or an alternative phone. The method only presumes there is an output PLY file representing a colored surface triangulation. An interesting alternative sensor that seems well-suited for this application is the Structure Sensor from Occipital (Boulder, CO), which was for example used in an EEG source localization study (Homölle and Oostenveld 2019).

### 4.3. Alternative belt designs and scenarios

Our belt, which uses particularly colored stickers placed at specific electrodes, could be easily modified to work with other belts. The codes in Murphy (2023a) are easily modifiable to account for different orders and symbols of known electrodes. Specifically, the belt and code could easily be modified to work with 16 electrode belts as opposed to 32 electrodes. Although it is outside the scope of this study, the code could likely be modified to handle scenarios where patients are ill (e.g. have ALS and cannot stand) or are unconscious patients on a ventilator. In these cases, one could likely (1) merge multiple sub-scans (e.g. a front and a back scan or left and right scan for a subject unable to stand) like we implemented in a similar study (Everitt *et al* 2023), or (2) the back-side could be smoothly interpolated for unconscious patients—alternatively, the EIT scenario could be adjusted to exclude dorsal electrodes (Park and Kwon 2024).

### 4.4. Comparison to other scanning approaches and boundary studies

The most similar study to this one is Dussel *et al* (2022), in which video recorded from a smartphone was used to render a thorax surface with electrodes identified and labeled. In comparison, this study used an iPhone with the Heges app to produce thorax surfaces (roughly real-time) with electrodes labeled quickly using custom Matlab software (Steps 1–2 in figure 1). Dussel *et al* validated on a mannequin. Thus, we see the study here yielded somewhat smaller errors but with a much smaller standard deviation (3.2 ± 0.4 mm from table 1 compared to 5.4 ± 6.0 mm) in a shorter period of time (3.4 min compared to 5.3 min). Additionally and importantly, this study rapidly produced full subject-specfic FEM meshes from the thorax surfaces (Step 3 in figure 1), which was not considered in Dussel *et al* (2022). In terms of other studies, the approach developed here can provide an initial accurate subject specific mesh that can be perturbed via inverse solutions (Soleimani *et al* 2006, Nissinen *et al* 2011, Brazey *et al* 2022) or belt/electrode motion (de Gelidi *et al* 2018, Darma *et al* 2020). Further, the software to rapidly produce FEM meshes could be incorprorated into these meshes, essentially allowing for easy construction of a set of meshes that are accurate through a full breathing cycle.

## 5. Conclusion

This study described an iPhone-based method to rapidly produce subject-specific FEM meshes (∼6 min from PLY files to a full 3D FEM mesh) with high degrees of precision (3.2 ± 0.4 mm RMSE) and accuracy (4.0 ± 0.3 mm RMSE) on mannequin validation tests and only slightly larger errors on volunteer data (5.2 ± 2.1 mm RMSE precision and 7.7 ± 2.9 mm RMSE accuracy). The codes are publicly available (Murphy 2023a, 2023b). Easy to generate subject-specific meshes could be utilized in the lung-EIT community, potentially reducing geometric-based artifacts and improving the clinical utility of EIT as a continuous, ambulatory pulmonary monitoring modality.

## Data Availability

The data that support the findings of this study are openly available at the following URL/DOI: https://zenodo.org/records/10514628.

## Ethical statement

The research was conducted in accordance with the principles embodied in the Declaration of Helsinki and local requirements. All subjects participating in the PFT aspect of the study were consented as part of an IRB approved study at Dartmouth Hitchcock Medical Center (study number 02000545). All data presented and made public has been deidentified by removing any names and cropping identifiable information.
